# A systematic review of the barcoding strategy that contributes to COVID-19 diagnostics at a population level

**DOI:** 10.3389/fmolb.2023.1141534

**Published:** 2023-07-11

**Authors:** Heng-Chang Chen

**Affiliations:** Epigenetics of Infectious Diseases Research Group, Center for Population Diagnostics, Łukasiewicz–PORT Polish Center for Technology Development, Wrocław, Poland

**Keywords:** barcoding technology, SARS-CoV-2, COVID-19, COVID-19 diagnostics, population diagnostics

## Abstract

The outbreak of SARS-CoV-2 has made us more alert to the importance of viral diagnostics at a population level to rapidly control the spread of the disease. The critical question would be how to scale up testing capacity and perform a diagnostic test in a high-throughput manner with robust results and affordable costs. Here, the latest 26 articles using barcoding technology for COVID-19 diagnostics and biologically-relevant studies are reviewed. Barcodes are molecular tags, that allow proceeding an array of samples at once. To date, barcoding technology followed by high-throughput sequencing has been made for molecular diagnostics for SARS-CoV-2 infections because it can synchronously analyze up to tens of thousands of clinical samples within a short diagnostic time. Essentially, this technology can also be used together with different biotechnologies, allowing for investigation with resolution of single molecules. In this Mini-Review, I first explain the general principle of the barcoding strategy and then put forward recent studies using this technology to accomplish COVID-19 diagnostics and basic research. In the meantime, I provide the viewpoint to improve the current COVID-19 diagnostic strategy with potential solutions. Finally, and importantly, two practical ideas about how barcodes can be further applied in studying SARS-CoV-2 to accelerate our understanding of this virus are proposed.

## 1 Introduction: the general principle of the barcoding strategy

**FIGURE 1:**
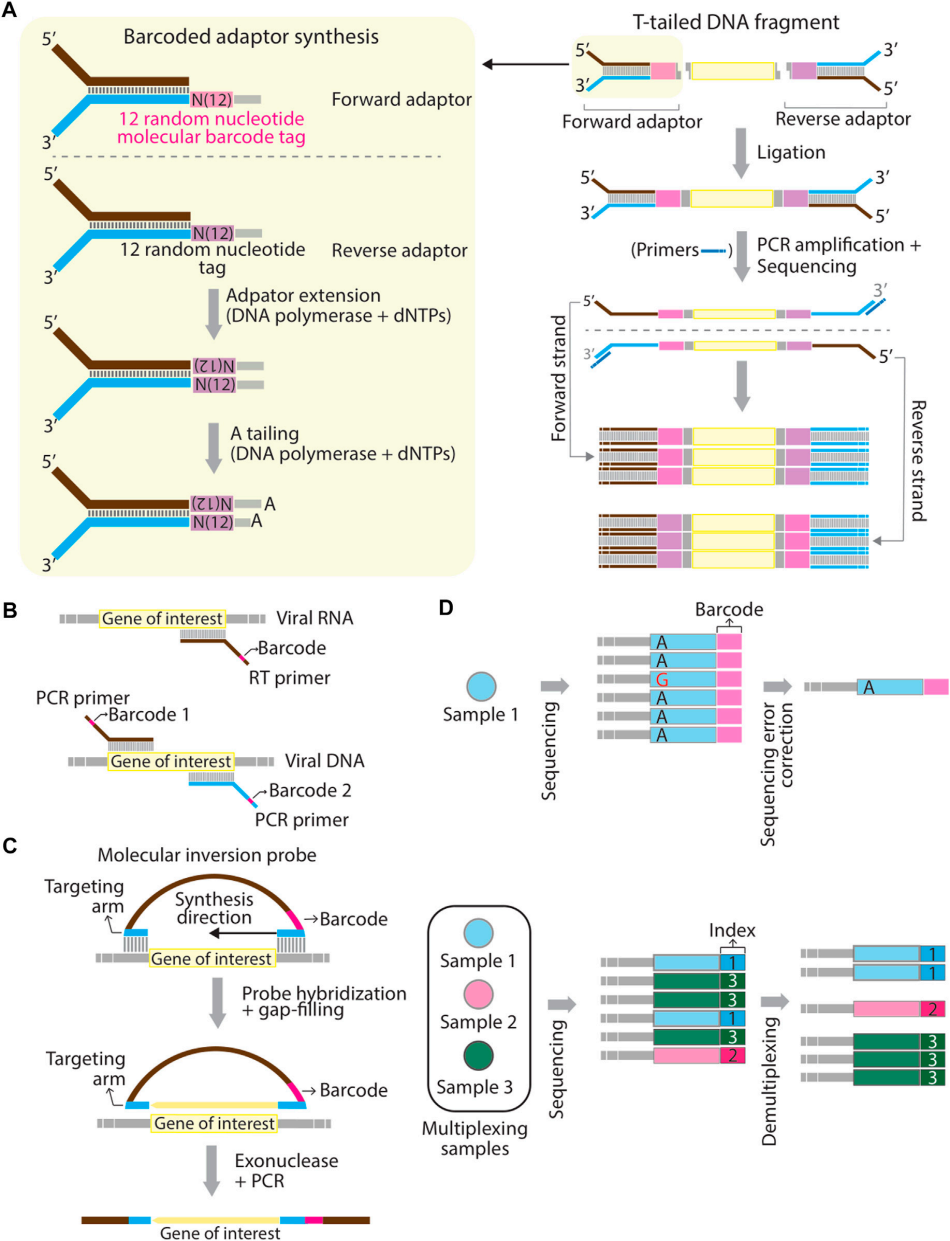
Schematic representation of mechanistic strategies of barcoding. **(A–C)** Barcodes can be introduced to a template using adaptors through direct ligation **(A)**, using RT- or PCR primers at the reverse transcription or PCR amplification step **(B)**, and using hybridizing molecular inversion probes **(C)**. **(D)** Schematic representation of the difference between “barcodes” and “sample indexes”. Barcodes aim to correct sequencing errors. For example, a misreading nucleotide, guanosine **(G)** can be corrected in final consensus sequences for a pool of Sample 1 (top panel). Sample indexes are used to multiplex different sequencing amplicons generated from different pools of samples (Sample 1, 2, and 3) (bottom panel). Panel **(A)** is modified based on Figure 1 in (Schmitt et al., 2012) and panel **(C)** is modified based on Figure 1 in (Hiatt et al., 2013).

Barcoding strategy has first proposed to solve the problems of PCR duplications and to improve the accuracy of next-generation sequencing quantification (Casbon et al., 2011; Kinde et al., 2011). In the past, barcodes have been given various names, such as unique molecular identifier (UMI) (Kivioja et al., 2012), primer ID (Jabara et al., 2011), and duplex barcodes. Barcodes are usually in the string form of random nucleotides, partially degenerate nucleotides, or defined nucleotides. The concept of the barcoding strategy is that individual original DNA or RNA fragments within the same pool of samples are tagged with a unique sequence of a molecular tag (Peng et al., 2015). Sequence reads that contain different barcodes illustrate different origins of molecules, whereas sequence reads with the same barcodes are the result of PCR duplication from the same original molecule (Peng et al., 2015). In general, the workflow of studies using barcoding technology consists of several main steps including 1) tag samples of interest with unique barcodes, 2) multiplex samples, 3) proceed barcoded samples by sequencers or other high-throughput techniques, and 4) demultiplex readouts and assign each sample to the corresponding barcode. Barcodes can be introduced in at least three ways. In the first approach, barcodes are embedded into molecular adaptors while constructing sequencing libraries. A classic example was given by (Schmitt et al., 2012). They first generated a pair of double-stranded and Y-shaped adaptors embedded with unique barcodes and ligated them to both ends of amplicons. This sequencing library is made to correct sequencing errors shown in sequencing reads (Figure 1A). Several commercial kits already provide the option of a PCR-free barcoding procedure with the same logistic strategy (so-called direct ligation approach shown in Table 1). In the second approach (so-called primer-associated approach in the following context and Table 1), barcodes are embedded in target-specific primers and introduced on a template by reverse transcription (RT) or PCR amplification (Figure 1B). The third approach is to use molecular inversion probes (MIP) carrying barcodes. A classic example was shown in the study from Hiatt et al. (2013) (Hiatt et al., 2013), where molecular tags (the same as barcodes discussed here) were introduced to the reverse complement strand of the gene of interest using polymerase and ligase, allowing distinguishing reads derived from different genomic equivalents within individual DNA samples (Figure 1C). Other methods that are not frequently used for barcoding will also be briefly discussed in the latter section (summarized in Table 1). The following contents will focus on barcoding applied to diagnostics of SARS-CoV-2 infections and biologically-relevant studies.

**TABLE 1:** Systematic comparison of barcoding strategies used in the category of molecular barcodes.

Approach	Type	Strategy	Combinations of different pairs of barcoding	PCR required for barcoding	Target SARS-CoV-2 gene(s)/protein(s)	Commercial kit used for barcoding	Sample type	Platform/Experimental pipeline	Required software^a^	Functions of barcodes	Major contribution of the technology	Ref.
Primer-associated approach	Sequence-based barcodes	SQK-RBK004: transposase carrying barcodes to the site of the cleavage	-	-	Whole genome	Oxford Nanopore Rapid Barcoding kit (SQK-RBK004)	SARS-CoV-2 patient samples (nasopharyngeal swab)	Oxford Nanopore	Guppy version 3.6.0; ARTIC Network bioinformatics protocol	Multiplex samples	Propose a method to sequence the whole genome of SARS-CoV-2 in a rapid and cost-efficient manner	Freed et al. (2020)
Protein-protein interaction approach	Sequence-based barcodes	A pair of DNA barcodes is installed on the SARS-CoV-2 spike protein S1 subunit	-	-	S	n/a	57 blood specimens	Hamilton Microlab ADAP STAR automated liquid-handling platform	PRISM v8.1.1; XLSTAT software 2019.1	Quantify protein-antibody interaction	Qualitative detection of total antibodies against S1 subunit of the spike protein	Karp et al. (2020)
CRISPR-associated approach	Sequence-based barcodes	Unique sgRNA sequences that serve as unique barcodes are encoded on the same plasmid used for making the dCas9-fusion library	-	-	S, N	n/a	6 COVID-19 convalescent samples	DNA microarray platform (GenePix 4300A microarray scanner)	Microarray data processing scripts (GitHub); GenePix Pro 7	Multiplex samples	Characterization of (polyclonal) antibody-epitope binding	Barber et al. (2021)
Primer-associated approach	Sequence-based barcodes	Tagmentation performed by bead-linked transposes and PCR amplification	-	+	ORF1ab, S, E, M, N	IDT for Illumina Nextera UD Indexes Set A, B, C, D (384 indexes, 384 samples)	752 patient sample (nasopharyngeal swab, oropharyngeal swab, and nasal swab)	Illumina NovaSeq 6000	Illumina DRAGEN COVIDSeq Test Pipeline	Multiplex samples	Population diagnostics (1536 sequencing libraries) and a confirmatory test; variants analysis	Bhoyar et al. (2021)
Primer-associated approach	Sequence-based barcodes	Barcodes embedded in the RT-PCR primers	+	+	S, N	n/a	Extraction-free lysates from mid-nasal swabs and saliva	Illumina MiSeq, MiniSeq and the Illumina NextSeq 550 systems	R package swabseqr	Multiplex samples	Population diagnostics (80,000 samples)	Bloom et al. (2021)
Primer-associated approach	Sequence-based barcodes	Barcodes embedded in the RT primers	+ (barcode and UMI)	+	N	n/a	Hospitalized patient samples (5 s of exhaled breath was collected in the Bubbler)	Bubbler breathalyzer	Bowtie software version 2.2.4	Multiplex samples	Direct detection of SARS-CoV-2 from exhaled breath	Duan et al. (2021)
Primer-associated approach	Sequence-based barcodes	SISPA† barcoding primers are given at the step of RT; Ligation of barcodes (Oxford Nanopore sample indexes) to both ends of DNA sequencing amplicons	+ (SISPA dual barcodes + Oxford Nanopore barcodes)	+	Whole genome	Native barcoding expansion 96 kit (EXP-NBD196)	43 clinical specimens (oropharyngeal swab and nasopharyngeal swab)	Oxford Nanopore	Guppy (Version 5.0.7, Oxford Nanopore Technologies); BugSeq (version 1.1, database version: RefSeq on 28 Jan 2021)	SISPA barcoding primers enable to detect and assemble genomes of SARS-CoV-2; Oxford Nanopore barcoding used for multiplexing samples	Variants analysis	Gauthier et al. (2021)
Primer-associated approach	Sequence-based barcodes	SQK-RBK004: transposase carrying barcodes to the site of the cleavage; no barcodes are used in the kit SQK-LSK109	-	-	Whole genome	The Rapid Barcoding kit (SQK-RBK004) and the Ligation Sequencing Kit (SQK-LSK109)	Clinical samples	Oxford Nanopore	Guppy version 4.0.11 (community.nanoporetech.com) and the high accuracy version of the flip-flop algorithm	Multiplex samples	Comparison of two kits used for the whole SARS-CoV-2 genome sequencing	González-Recio et al. (2021)
Primer-associated approach	Sequence-based barcodes	LAMP barcodes embedded in the forward inner primer (FIP); PCR barcodes (indexes, i5 and i7) introduced at the PCR stage	+ (LAMP-barcodes + PCR-barcodes)	+	E, N	n/a	SARS-CoV-2 swab samples	Illumina MiSeq or iSeq sequencer	LAMP-Seq Inspector so ware (http://manuscript.lamp-seq. org/Inspector.htm)	LAMP barcodes: specify each sample; PCR barcodes: multiplex samples	Population diagnostics (676 samples)	Ludwig et al. (2021)
Direct ligation approach	Sequence-based barcodes	Unique barcodes are generated by combining different A and B oligos	-	-	Whole genome	n/a	PBMCs from 18 SARS-CoV-2-infected patients	Ion Torrent PGM 314 or 316 chip (Life Technologies)	‘Barracoda’ (https://services.healthtech.dtu.dk/service.php?Barracoda- 1.8)	Multiplex samples	Identification of immunogenic CD8^+^ T cell epitopes	Saini et al. (2021)
Primer-associated approach	Sequence-based barcodes	The first barcode is introduced in the primer for reverse transcription; the second barcode is given in the step of PCR amplification. This strategy is called “Concat-PCR”	+ (the first- and the second barcodes)	+	E, S	Oxford Nanopore PCR Barcoding Expansion 1–96 kit (EXP-PBC096)	SARS-CoV-2 synthetic RNA	Oxford Nanopore	RETIVAD	Multiplex samples	Diagnostics (proof of concept); variants analysis	Stüder et al. (2021)
Staining approach	Color-based barcode	106 Ramos B cells are resuspended in different concentrations of the cell proliferation tracer CytoTell blue	-	-	S, RBD	n/a	12 COVID-19 patient samples	Color-based barcoded spike protein flow cytometric assay (BSFA)	n/a	Label and separate samples	Comparison of immune responses triggered by different variants of SARS-CoV-2	Vesper et al. (2021)
Primer-associated approach	Sequence-based barcodes	Two unique barcodes embedded in primers at the stage of RT	+ (the left and the right barcodes)	+	S	n/a	Commercial pooled human saliva from healthy individuals with spiked-in synthetic viral RNA	INSIGHT [isothermal NASBA (nucleic acid sequence–based amplification) sequencing–based high- throughput test]; Illumina MiSeq (PE 150bp)	FASTX_trimmer	Multiplex samples	Diagnostics (48 samples)	Wu et al. (2021)
CRISPR-associated approach	Sequence-based barcodes	Customized peptide libraries are designed to encode a unique 20 bp nucleic acid sequence used as the gRNA barcode	-	-	S	n/a	COVID-19 samples (convalescent, pre-vaccine and post-vaccine)	Cas9 display (CasPlay) system (GenePix 4300A microarray scanner); Illumina NextSeq 500 (single-end 150 bp) used to sequenced dCas9-fusion library	GenePix Pro 7; Cutadapt v2.5 Martin. (2011) and customized commend lines	Multiplex samples	Evaluation of vaccine-induced antibody reactivities from the SARS-CoV-2 proteome	Barber et al. (2022)
Primer-associated approach	Sequence-based barcodes	Barcodes embedded in the forward primer during the step of one-step-RT-PCR	-	+	ORF1, E, N1	n/a	960 Oro- and nasopharyngeal swabs collected from SARS-CoV-2 patients	Illumina Miseq sequencer (PE150 bp)	R package DNABarcodes created unique ten-base barcodes; FASTX-toolkit version 0.0.14 (http://hannonlab.cshl.edu/fastx_toolkit/)	Multiplex samples	Variants analysis	Cohen-Aharonov et al. (2022)
Primer-associated approach	Sequence-based barcodes	A barcode is introduced upstream of the ribosome-binding site in the recombined pDEST–MIPSA vector	-	+	n/a	n/a	55 COVID-19 patient samples	MIPSA (Molecular Indexing of Proteins by Self-Assembly); Illumina MiSeq	The MIPSAlign package (https://github.com/jgunn123/MIPSAlign)	Individually label 12,680 human clonal open reading frames (mapped to 11,437 genes)	Identification of autoreactive antibodies in plasma samples	Credle et al. (2022)
Protein-protein interaction approach	Sequence-based barcodes	DNA barcodes are cross-linked on the S1 subunit protein and the ACE2 receptor	-	-	S1	n/a	146 COVID-19 serum samples	Split-Oligonucleotide Neighboring Inhibition Assay (SONIA) on the basis of real-time qPCR	n/a	Detect whether neutralizing antibodies block the binding between the S1 protein and the ACE2 receptor	Quantification of neutralizing antibodies binding on SARS-CoV-2 S protein subunit 1	Danh et al. (2022)
Primer-associated approach	Sequence-based barcodes	Ligation of barcodes to both ends of DNA of interest after performing FFPE repair and end-prep	-	-	Whole genome and the spike region of interest (positions 23,468 to 23,821)	Native Barcode expansion kit (EXP-NBD196); Ligation sequencing kit (SQK-LSK109)	The hamster and pneumocyte samples	Oxford Nanopore	MinKNOW v4.3.7.; Guppy 5.0.12	Multiplex samples	Variants analysis	Escalera et al. (2022)
Direct ligation approach	Sequence-based barcodes	A barcode is inserted in a string of the DNA sequence used to link to biotinylated spike ectodomain or spike-RBD	-	-	S (spike ectodomain and RBD)	Solulink Protein-Oligonucleotide Conjugation Kit (TriLink cat no. S-9011)	Mice models (BALB/c and C57BL/6J)	LIBRA-seq technology; single-cell RNA sequencing (10x Genomics User Guide, CG000186 Rev D)	CITE-seq-Count Mimitou et al. (2019)	Barcode on each B cell to indicate its antigen specificity	Identification of an antibody (SW186) that can neutralizes SARS-CoV-2	Fang et al. (2022)
Primer-associated approach	Sequence-based barcodes	Spike-ins barcodes are prepared according to the PrimalSeq v.4.0 protocol Matteson et al. (2020)	+ (Spike-ins barcodes + NEXTflex Dual-Indexed DNA Barcodes)	+	Whole genome	n/a	49 SARS-CoV-2 patient samples	Illumina MiniSeq (PE150 bp)	Cutadapt v.2.10 (demultiplexing) Martin. (2011); iVar Grubaugh et al. (2019)	Spike-ins barcodes used to detect potential sample cross-contamination; indexed DNA barcodes: multiplexing samples	Reconstruction of SARS-CoV-2 transmission history	Gallego-García et al. (2022)
Antibody-staining approach	Sequence-based barcodes	Cells were incubated with mixtures of barcoded antibodies	-	-	n/a	TotalSeq^TM^-A Antibodies and Cell Hashing with 10x Single Cell 3′ Reagent Kit v3 3.1 Protocol (Biolegend)	Clinical PBMC samples from SARS-CoV-2 patients (277 TotalSeq-A antibodies)	TotalSeq antibodies in combination with single-cell RNA sequencing (Illumina NovaSeq S4 flow cell)	Cell Ranger 3.1.0 with default parameters ( https://github.com/10xGenomics/cellranger)	Multiplex samples	Benchmark different hashing methods for single-cell RNA-seq on clinical samples from SARS-CoV-2 patients	Mylka et al. (2022)
Primer-associated approach	Sequence-based barcodes	Barcodes embedded in primers to perform first-strafed cDNA	+ (A well-specific barcode and a plate-specific barcode)	+	N	Illumina indexed barcoding kit (optional)	4 SARS-CoV-2 patient samples	Reombinase mediated barcoding and amplification diagnostic tool (REMBRANDT) associated with Illumina sequencing platform (MiSeq)	The REMBRANDT pipeline ( https://github.com/MilesLab/Rembrandt_pipeline/)	Specify samples in each well and in each plat and multiplex samples for sequencing	Scalable diagnostic test (6 samples tested, 4 SARS-CoV-2-positive, 2 SARS-CoV-2-negative)	Palmieri et al. (2022)
Primer-associated approach	Sequence-based barcodes	Same design described in LAMP-Seq Ludwig et al. (2021)	+ (LAMP-barcodes + PCR-barcodes)	+	N	n/a	Contrived saliva samples and 120 clinical nasopharyngeal swab samples	COV-ID pipeline: RT-LAMP combined with Illumina NextSeq or similar instrument	FASTX-toolkit utility fastq_quality_ lter ( http://hannonlab. cshl.edu/fastx_toolkit/); Cutadapt Martin. (2011)	See LAMP-seq Ludwig et al. (2021)	Scalable diagnostic test and an approach for the simultaneous detection of different pathogens, including SARS-CoV-2 in contrived saliva samples	Warneford-Thomson et al. (2022)
Primer-associated approach	Sequence-based barcodes	Patient barcodes (10 bp) introduced during the initiation of the RT step; plate barcodes given at the PCR step	+ (Patient barcode and plate barcode)	+	Whole genome^‡^	n/a	Synthetic RNA templates of SARS-CoV-2; 45 patient samples (nasopharyngeal swabs and saliva samples)	DeepSARS with the Illumina MiSeq system (2 × 81 and 1 × 150 cycle runs)	R package Rsubread	Multiplex samples	Scalable diagnostic test; variants analysis	Yermanos et al. (2022)
CRISPR-associated approach	Sequence-based barcodes	External barcodes (4 bp) added at the external region outside of sgRNA at its 3′end	-	-	S	n/a	n/a	High-throughput sequencing (not specified in this study)	eBAR-analyzer (https://github.com/wolfsonliu/FluorescenceSelection)	Execute a high multiplicity of infection (MOI) in generating the cell library for screening	Identify novel host factors required for SARS-CoV-2 entry	Zhu et al. (2022)

^a^ Software indicated here are those used to proceed with raw sequencing reads.

^†^Sequence-independent single primer amplification (SISPA) (Reyes and Kim, 1991).

^‡^The whole genome sequence was obtained based on a multiple sequence alignment of short reads (110–140 bp) sequenced by Illumina MiSeq.

## 2 Subsections

### 2.1 Background information about SARS-CoV-2 and COVID-19

The outbreak of novel coronavirus disease 2019 (COVID-19) caused by severe acute respiratory syndrome coronavirus 2 (SARS-CoV-2) occurred in early December 2019 and has quickly spread worldwide and turned into a global pandemic. Although the origin of SARS-CoV-2 has been the topic of substantial debate (a natural origin through zoonosis or the introduction from a laboratory source), molecular evidence indicates that coronaviruses originated in bats (Drexler et al., 2014) and then transmitted to civets and several wildlife species as potential intermediate hosts, and then to humans. Coronaviruses, like other RNA viruses that can frequently undergo host switching under different selection pressures, are genetically heterogeneous, in part due to the highly error-prone and low-fidelity RNA-dependent RNA polymerases that replicate their genomes (Vignuzzi et al., 2006; Peck and Lauring, 2018; Jones et al., 2021), resulting in this virus possibly infecting a broad spectrum of hosts.

The genome of SARS-CoV-2 is composed of 29,881 nucleotides (Lu et al., 2020), making this virus one of the largest known single-stranded RNA-enveloped viruses. Its genome encodes four structural proteins, including spike (S), small protein (E), matrix (M), nucleocapsid (N) (Chan et al., 2020), and other accessory or non-structural proteins. In SARS-CoV-2, the S protein is the main structural protein to ensure the attachment of the virion to the target cell and mediate membrane fusion, thereby achieving successful viral entry (Ou et al., 2020) and being a key protein in determining the infectivity of this virus and the transmissibility in the host (Hulswit et al., 2016). Additionally, this protein is also the major antigen inducing protective immune responses (He et al., 2004; Du et al., 2009; Li, 2016; Walls et al., 2020). Pathologically speaking, it is suggested that severe COVID-19 results from virus-driven perturbations in the immune system and tissue injury, including neutrophil extracellular traps, and thrombosis even though the mechanisms that lead to manifestations of viral infection are not fully understood.

### 2.2 Literature search strategy

A rigorous literature search was done using PubMed with the keywords ((SARS-CoV-2) OR (COVID-19)) AND (barcode). Research articles were searched from 2019 till the time of writing (end of November 2022), with the limitation of solely selecting the research articles published in the English language and the exclusion of the review articles and preprints, and news features. In the first place, 345 articles were released from PubMed searching with the keywords. A careful examination was then performed throughout all articles and removed the ones that do not match the scope of this Mini-Review. Eventually, 45 articles fit the criteria. Based on the function and the type of barcodes described in these 45 articles, the barcodes were classified into three categories: molecular barcodes (26 articles), genetic barcodes (10 articles), and digital barcodes (9 articles). Molecular barcodes refer to sequence-based barcodes, which are often implemented with different biotechnologies, such as PCR, RT-PCR, flow cytometry, CRISPR/Cas9 and so on. In contrast, in this Mini-Review genetic barcodes refer to either the unique viral genomic regions, enabling to classify SARS-CoV-2 variants or host cellular genetic signatures (Fischer et al., 2021). It is worth noting that although the concept of viral genetic barcodes is indeed fascinating for tracking and discriminating variants of SARS-CoV-2 and perhaps can also be beneficial for COVID-19 diagnostics, computational methods/algorithms used to retrieve genetic barcodes are presently not optimized and how frequently that currently known genetic barcodes still remain in the latest variant of SARS-CoV-2 is required to be evaluated. Here I summarize sequences of known genetic barcodes present in major clades, and their corresponding variants, and SARS-CoV-2 genes in Table 2. Genetic barcodes were collected from (Guan et al., 2020) and Zhao et al. (2020) (Zhao et al., 2020). Digital barcodes refer to 2D QR barcodes used to store information. In this Mini-Review, the focus will be placed on molecular barcodes. A comparison of the articles using molecular barcodes is summarized in Table 1.

**TABLE 2:** Known genetic barcodes present in main clades of SARS-CoV-2.

Clade	Variant	Corresponding SARS-CoV-2 gene	Genetic barcode sequence^a^
A1	G26144T	ORF3a	CCCGCCAAGtG
A1a	G11083T	ORF1ab	CCCttCAAGtG
	C14805T	ORF3a	
A2	C241T	5′UTR	CCCGCAGgGGG
	C3037T	ORF1ab	
	A23403G	S	
A2a2	G25563T	ORF3a	CCCGCCAgtGG
A2a2a	C1059T	ORF1ab	tCCGCCAgtGG
A2a4	G28881A	N	CCCGCCAgGGa
	G28882A	N	
	G28883C	N	
A2a5	A20268G	ORF1ab	CCCGCCggGGG
A3	G11083T	ORF1ab	CCCtCCAAGGG
B	C8782T	ORF1ab	CCtGCCAAGGG
	T28144C	ORF8	
B1	C18060T	ORF1ab	CCtGCtAAGGG

^a^ Lowercase letters shown in genetic barcodes represent the nucleotide that differ from the SARS-CoV-2, reference sequence (MN908947.3).

### 2.3 The barcoding strategy for studying SARS-CoV-2

Barcodes used in these 26 articles are sequence-based, except the study from (Vesper et al., 2021), in which the authors used different concentrations of the cell proliferation tracer, CytoTell blue, as color-based barcodes read by the flow cytometry. The barcoding step can be achieved either using commercial kits, like the kits from Illumina and Oxford Nanopore Technologies, or a customized design (Table 1). In the latter case, a sequence of a barcode is often embedded in primers as an overhang at the step of reverse transcription of viral RNA or PCR amplification of the RT product (Figure 1B) (Bhoyar et al., 2021; Bloom et al., 2021; Duan et al., 2021; Gauthier et al., 2021; Ludwig et al., 2021; Stüder et al., 2021; Wu et al., 2021; Cohen-Aharonov et al., 2022; Credle et al., 2022; Gallego-García et al., 2022; Palmieri et al., 2022; Warneford-Thomson et al., 2022; Yermanos et al., 2022). Barcoded primers used in SwabSeq (Bloom et al., 2021) and by Cohen-Aharonoc et al. (2022) (Cohen-Aharonov et al., 2022) are compatible with one-step RT-PCR. The workflow of barcoding here resembles the primer-associated approach described in the first section that yields a final product of the amplicons carrying barcodes when the procedure of RT-PCR is complete. The length of barcodes can vary (generally between 4–20 base pairs): the longer length of a barcode is, the lower probability that multiple reads contain the same barcode. Of note, “barcodes” and “sample indexes” are conceptually two different molecular tags even though they both consist of a string of a DNA sequence. There are indeed some functional overlaps. However, precisely speaking, “barcodes” resolve to correct sequencing errors, thereby increasing sequencing accuracy, whereas “sample indexes” are used to multiplex sequencing libraries into the same lane of flow cells (Figure 1D). It is noteworthy that while reviewing these articles, I notice that it presently appears to be ambiguous between the usage of the term “barcodes” and “sample indexes”.

Using a combination of multiple barcodes or different layers of barcoding appears to be popular to increase the sequencing capacity and make the readouts more informative. For example, amplicons from SwabSeq (Bloom et al., 2021) are subjected to barcodes (i5 and i7) used to maximize the specificity and avoid false-positive results. Amplicons from LAMP-Seq (Ludwig et al., 2021) and COV-ID (Warneford-Thomson et al., 2022) contain one LAMP barcode (10 bp used in LAMP-Seq and 5 bp used in COV-ID) and two standard PCR barcodes (Illumina i5 and i7) to scale up the deep sequencing capacity. Gauthier et al. (2021) (Gauthier et al., 2021) employed SISPA barcoded primers (Reyes and Kim, 1991) to detect and assemble genomes of SARS-CoV-2 and Oxford Nanopore barcodes to multiplex samples. Stüder et al. (2021) used two sets of barcoded primers to track variants of viruses and multiplex samples for sequencing. Wu et al. (2021) embedded a left and a right barcode (5 bp of each) in the forward- and the reserve primer, respectively, to specify patient samples. Gallego-García et al. (2022) spiked in a string of 20 random nucleotide barcode sequences inserted in the forward- and reverse primer to minimize cross-sample contamination. Palmieri et al. (2022) and Yermanos et al. (2022) applied two-dimensional barcoding primers to specify samples pooled in wells and plates. Similarly, Duan et al. (2021) directly included a known sequence of a barcode (8 bp) and a UMI with three random nucleotides in RT primers at the same time to multiplex samples and correct sequencing reads. In addition to the PCR- or RT-PCR-based method, barcodes can also be introduced using different ways. For example, Danh et al. (2022) used chemical cross-linkers to install DNA barcodes. Studies from Fang et al. (2022), Saini et al. (2021), and Karp et al. (2020)) directly ligated a DNA sequence of a barcode to the protein of interest (peptide-MHC complex multimers or the spike protein), which is a PCR-free approach. Mylka et al. (2022) used barcoded-labeled antibodies or lipid anchors to stain a pool of cells individually. More importantly, the spectrum of its application can be broadened when barcoding is adapted to other biotechnologies. For example, designed unique sgRNA to serve as identifiers (unique barcodes), which are co-expressed with the Cas9 protein Barber et al. (2021); Barber et al., 2022). Zhu et al. (2022) included an additional sequence-based barcode adjacent to the 3’ end of sgRNA in addition to unique guide sequences. As mentioned previously, Vesper et al. (2021) applied the cell proliferation tracer dye with different dilutions to label samples, allowing samples to be separated using flow cytometry.

### 2.4 The barcoding strategy for current COVID-19 diagnostics, fundamental research, and future perspectives

One of the main contributions of barcoding is to scale up testing capacity for population diagnostics. Diagnostics at a population level has become one of the essential strategies to control the outbreak of COVID-19 because it allows the detection of people with SARS-CoV-2 infections in the first place and immediately places them in quarantine. Available and mature methods, which have been benchmarked for COVID-19 diagnostics at a population level include DRAGEN COVIDSeq (Bhoyar et al., 2021), SwabSeq (Bloom et al., 2021), and LAMP-Seq (Ludwig et al., 2021) (Table 1). Amplicons prepared based on these methods are sequenced using Illumina sequencing platforms (iSeq, MiniSeq, MiSeq, NextSeq, NovaSeq). One strong advantage of Illumina sequencing is that Illumina adapter sequences are made public, benefiting researchers to implement barcodes adapted to their experimental designs subtly. These methods are made to diagnose a small region of a gene, thereby shortening the duration of diagnostic time. Most importantly, these methods appear to be less labor-intensive and cost-effective. Other potential methods for COVID-19 population diagnostics are listed in Table 1. Although nowadays public health policy in many countries tends to coexist with viruses, COVID-19 diagnostics is still crucial to control the spread of the disease in countries where medical resources are insufficient. Since around 33% of people with SARS-CoV-2 infection are estimated to be asymptomatic Oran and Topol (2021) the accurate assessment of COVID-19 diagnostic capacity remains important in first place for strategic planning, public health control measures, and patient management.

In addition to multiplexing samples, several groups apply barcoding to identify new variants of concern (Bhoyar et al., 2021; Gauthier et al., 2021; Stüder et al., 2021; Cohen-Aharonov et al., 2022; Escalera et al., 2022; Gallego-García et al., 2022; Yermanos et al., 2022) (Table 1). Indeed, SARS-CoV-2 is a typical zoonotic RNA virus that enables itself to complete infection across different species. The appearance of viruses that evolve to adapt to a new living niche often reflects on viral sequence changes. Fixation of these changes may require a long time through repeated transmission, eventually resulting in a reduced size of an effective population harboring dominant alterations in their sequence spaces. Investigation of how the virus genetically evolves to achieve host jumps could therefore be essential to understand the molecular basis of this process, benefitting developing better antiviral strategies. One of the methodologies to study virus cross-species transmission is to use the reverse genetics approach, allowing elucidation of the consequence of genetic mutations by examining changes to phenotypes. Here, I propose that barcodes could be implemented in the *in vitro* or *in vivo* system and used as tracers for reconstructing individual evolutionary transmission routes over a large experimental timescale. Practically, unique barcodes could be used to tag the genome of SARS-CoV-2 or embedded in SARS-CoV-2 pseudotyped virus. Barcoded viruses then infect an appropriate model system with multiple rounds of infection. Since barcodes distinguish individual viral infections, it becomes feasible to monitor the genetic alteration of individual viruses from different lineages of evolutionary paths.

Barcoding has also been applied to characterize specific antibody-epitope binding (Karp et al., 2020; Barber et al., 2021; Saini et al., 2021; Vesper et al., 2021; Barber et al., 2022; Credle et al., 2022; Danh et al., 2022; Fang et al., 2022), and identify novel host factors required for viral entry (Zhu et al., 2022) (Table 1). A typical feature of an RNA virus is high rates of mutations due to the high error-prone and low fidelity of the RNA-dependent RNA polymerase, thereby exhausting our immune system and weakening the efficacy of antiviral drugs. For this reason, an effective strategy to develop a broad spectrum of SARS-CoV-2 neutralizing antibodies and antiviral drugs that cover variants of SARS-CoV-2 is a requisite shortly. Another idea proposed here is to high-throughput select drug-resistant variants of SARS-CoV-2 *in vitro*. Barcoded SARS-CoV-2 will be used for *in vitro* infections in the presence of different antiviral drugs. After multiple rounds of infections, barcoded viruses that remain vivid are collected and sequenced. Based on unique barcodes, it thus becomes possible to unveil mutations, which are essential to resist the killing of corresponding antiviral drugs with resolution of individual viruses at a single-nucleotide level.

## 3 Discussion

In the past 10 years, technological progress in barcoding has been made to reach the resolution at a single-molecule level and detect low-frequency and subclonal variations. Such advantages are now applied to elevate diagnostic capacity and study or track variations of individual viruses in a pool of samples. Collectively, the advantages of barcoding strategy toward COVID-19 diagnosis include 1) increasing the throughput of diagnostic samples, 2) shortening the processing time, 3) diminishing the risk of technical batch effects, 4) lowing library preparation costs and per-sample cost, and 5) increasing accuracy of diagnostic results. Furthermore, the potential application of the barcoding strategy in SARS-CoV-2 research can be extended to track variants over a large timescale and perform SARS-CoV-2 progression surveillance beyond the usage in COVID-19 diagnosis. Nevertheless, critical issues (shortcomings of barcoding strategy), such as barcode collisions and barcode hopping are still required to pay attention. These problems could be solved at the experimental- and analytical level. The potential solution worked out at the bench could be by increasing the complexity of unique barcodes in a pooled library, thereby minimizing the probability that multiple molecules initially receive the same barcode (barcode collisions) or barcodes are incorrectly assigned to other molecules (barcode hopping) at the amplification step. The complexity of barcodes can be lifted either by increasing the abundance of a pool of barcodes (quantity) or adjusting a minimum Levenshtein distance (Yujian and Bo, 2007) among barcodes (quality). Alternatively, errors could also be corrected using better error-correcting algorithms and quantification methods.

In this Mini-Review, recent 26 research articles using the barcoding strategy, which mainly contributes to COVID-19 diagnostics and biological research of SARS-CoV-2 were systematically reviewed (Table 1). In addition to increased diagnostic capacity, rapid duration of diagnostic time, and low costs, the accuracy of diagnostic results is another factor that should be well considered. Several of the studies (Bloom et al., 2021; Gauthier et al., 2021; Ludwig et al., 2021) reviewed here already discussed and proposed possible solutions to correct false-positive results caused by barcode swapping. Importantly, it has been documented that up to 58% of COVID-19 patients may face initial false-negative diagnostics results (Pecoraro et al., 2022). One of the risks is due to frequent mutations in the genome of SARS-CoV-2, rendering primers used for detecting viruses ineffective. A potential solution could be to perform population-scale long-read sequencing. Although this idea has been put forward (Freed et al., 2020; Gauthier et al., 2021; González-Recio et al., 2021; Stüder et al., 2021; Escalera et al., 2022), the current methods are required to be further optimized. Essentially, two practical ideas to expand the power of barcoding are proposed in this Mini-Review. In the first idea, barcodes can be used as a tracer to depict the history of genome alterations in every lineage of variants of SARS-CoV-2 over time. It is beneficial to screen potential mutations that are required for cross-species transmission. The second idea would then benefit medical doctors to adjust antiviral regimens for treatments to satisfy the need of individual patients. Collectively, barcoding is one of the molecular tools that assist to read a massive array of samples in parallel and the onset of investigating variations at a population level.
